# The Interactive Effect of Goal Attainment and Goal Importance on Acculturation and Well-Being

**DOI:** 10.3389/fpsyg.2020.00704

**Published:** 2020-04-21

**Authors:** Agnes Toth-Bos, Barbara Wisse, Klara Farago

**Affiliations:** ^1^Department of Psychology, University of Groningen, Groningen, Netherlands; ^2^Department of Psychology, Eötvös Loránd University, Budapest, Hungary; ^3^Business School, Durham University, Durham, United Kingdom

**Keywords:** goal attainment, goal importance, intrinsic goals, migration, acculturation, well-being

## Abstract

The purpose of the present research is to shed light on whether and when migrants’ goal pursuit relates to their acculturation and well-being. Previous research has demonstrated the beneficial role of striving for and attaining intrinsic goals on well-being. Yet, the relationship between the pursuit of intrinsic goals and acculturation has hardly been addressed. To fill this void, we investigated whether migrants’ acculturation and well-being can be seen as a function of their pursuit of intrinsic goals. We posited that the attainment of intrinsic goals would positively predict migrants’ level of acculturation and subsequent well-being, particularly when migrants deemed these goals to be important. We tested our hypotheses in two scenario studies and two surveys. In all four studies we confirmed our hypothesis that migrants’ intrinsic goal attainment and well-being is mediated by their acculturation level. However, in only two of the four studies did we find support for our hypothesis that the relationship between intrinsic goal attainment and acculturation is moderated by intrinsic goal importance. We discuss the theoretical implications and the practical consequences of our findings. Furthermore, we outline future research directions that could deepen our understanding of the relationship between migrants’ goal pursuit and their acculturation.

## Introduction

**FIGURE 1:**
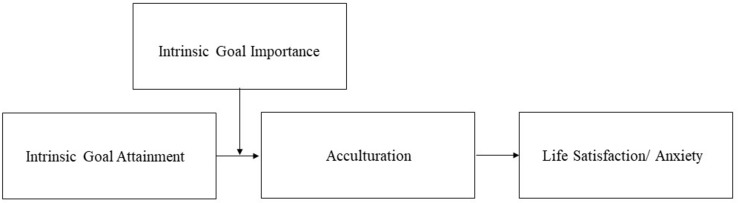
The proposed conceptual model of the effects of goal attainment on acculturation and life satisfaction and anxiety.

Rates of international migration have reached unprecedented levels throughout the world (International Organization of Migration [IOM], 2018). In 2017, 3.4% of the world’s inhabitants were international migrants, and an estimated 14% of people residing in high-income countries were migrants (United Nations, 2017). These migrants include not only refugees or asylum seekers – groups of migrants that have been in the spotlight lately – but also self-initiated migrants who choose to move to another country in order to pursue goals that are important to them. Many migrants, including those who have moved voluntarily to a new country, have difficulty finding happiness in their host country because adapting to the new situation is often difficult (Zheng et al., 2004; Hendriks, 2015). With migrant well-being a source of concern, it is not surprising that scholarly interest in acculturation, a main determinant of migrant well-being, has been increasing (Schwartz et al., 2013; Berry and Hou, 2016). The current study focuses on how migrant goal pursuit affects their acculturation level and, subsequently, their well-being. Only a few researchers have investigated goal pursuit in relation to adaptation or acculturation (Sheldon and Houser-Marko, 2001; Chirkov et al., 2007). In this paper, we use a self-regulation and goal pursuit perspective to argue that the attainment of goals that satisfy basic innate psychological needs may help migrants to feel more acculturated and happy, particularly when these migrants place a lot of value on these goals (see Figure 1 for our research model). As such, we point to the interplay of intrinsic goal attainment and intrinsic goal importance as an important precursor of acculturation and subsequent well-being. Our study may provide more insight into the factors that foster migrant acculturation and happiness and that could be used in interventions focused at increasing migration success. Finally, this paper combines experimental studies with online surveys using various measures and methods to analyze key concepts. This approach may strengthen the validity of the research model through cross-validation and the convergence of information from different sources.

## Acculturation and Well-Being of Migrants

Although migrants often leave their home country in an attempt to improve their lives, many migrants face unexpected challenges and stressful situations that are difficult to cope with (Sam and Berry, 2006). This makes migrants vulnerable in terms of mental health (Ward et al., 2001; Bhugra and Becker, 2005). Previous research has shown that migration is often associated with elevated psychosomatic problems (Carballo et al., 1998; Al-Baldawi, 2002), reduced well-being (Liebkind and Jasinskaja-Lahti, 2000), elevated depression levels (Bhugra, 2004), and increased drug and alcohol use (Polednak, 1997; Caetano and Clark, 2003). Apart from being detrimental to the migrants themselves, these adverse effects on mental health related outcomes also have negative downstream consequences for existing spouses and family members of migrants, for potential organizations that have hired these migrants, and for society at large (Mäkelä and Suutari, 2013; Selmer and Lauring, 2014; McNulty, 2015). Therefore, if acculturation improves migrant happiness this would be an important issue (Marsiglia et al., 2013).

Acculturation refers to all the changes that flow from the contact between individuals of different cultural backgrounds (Berry, 1997). Most researchers agree that acculturation is a dynamic, reciprocal process between migrant individuals or groups and host nationals (Berry, 1997; Ozer, 2017) upon which affective and behavioral changes take place in both parties (Trimble, 2003; Sam and Berry, 2006). Moreover, migrants’ acquisition of the beliefs, values, and practices of the host country does not automatically imply that they have discarded or rejected the beliefs, values, and practices of their country of origin (e.g., Berry, 1980). Indeed, host-culture acquisition and home-culture retention may be seen as independent dimensions.

In an ideal scenario, psychological acculturation results in both the psychological and sociocultural adjustment of the individual (Searle and Ward, 1990) in the country of settlement.

Psychological adaptation refers to how content and comfortable the individual feels in the changed cultural context (Demes and Geeraert, 2014). Sociocultural adaptation refers to a person’s ability to fit into the new culture and entails the practical and behavioral aspects of the adjustment (Ward and Kennedy, 1994, 1996). Given the psychological and cultural changes associated with the acculturation process, scholars have been focusing on the stressful nature of acculturation and investigated how acculturation may affect migrant well-being (Miller et al., 2011). The assumption is that acculturation is not an easy process and that low levels of acculturation may lead to emotional and psychological problems because it signals a lack of acclimatization to the new culture. As indicators of successful psychological acculturation, both psychological and sociocultural adjustment have been linked to greater well-being and lower levels of depression in migrants (Miller et al., 2011; Demes and Geeraert, 2014; Hirai et al., 2015). Moreover, a 2013 meta-analysis showed that acculturation revealed favorable relations to the entire spectrum of mental health, including both negative mental health and positive mental health indicators (Yoon et al., 2013).

Because acculturation is of major importance for well-being, researchers have previously studied the determinants of successful acculturation. These studies shed light on the impact of various demographic characteristics on acculturation, such as ethnic background (Phinney et al., 2001; Liebkind, 2006), socioeconomic status (Fitzgerald, 2010), language proficiency (Marsiglia et al., 2010), and country of origin (International Organization of Migration [IOM], 2013). Furthermore, researchers have studied the impact of individual and personality differences on acculturation, such as bicultural identity (Dion and Dion, 2006), cross-cultural competence (Chiu et al., 2013), coping styles (Kuo, 2014), and personal characteristics (Boneva and Frieze, 2001). Researchers have also provided rich information on the role of relevant social factors in acculturation, such as social support (Sullivan and Kashubeck-West, 2015) and discrimination (Torres et al., 2012). Interestingly, despite the fact that the migration process is often set in motion when people attempt to maximize their goal potentials, individual-level goal pursuit in relation to acculturation is largely understudied. We argue, however that the attainment of important goals may contribute to how rooted and embedded migrants feel in the host culture and whether they feel at home. Therefore, a self-regulation and goal-pursuit perspective could enhance our understanding of migration.

### Goal Pursuit, Acculturation, and Well-Being

Goals are future-oriented internal representations of desired states (outcomes, events, or processes) that a person strives to attain (Austin and Vancouver, 1996; Milyavskaya and Werner, 2018). According to self-determination theory (Deci and Ryan, 2000) two types of life goal can be distinguished, namely intrinsic and extrinsic goals (Kasser and Ryan, 1996, 2001). Intrinsic goals are those involving personal growth, loving relationships, health, and community service (Kasser and Ryan, 1996, 2001). Intrinsic goals are hypothesized to emerge from natural growth tendencies, in which individuals move toward expanded self-knowledge and deeper connections with others and the community, and are considered to be consistent with basic human nature and needs (Deci and Ryan, 2000; Kasser and Ryan, 2001). In contrast, extrinsic goals include financial success, physical attractiveness, and social fame and/or popularity. These goals are hypothesized to be strongly shaped by culture, usually involving symbols of social status and other people’s positive evaluation. These goals are considered to be less consistent with fundamental human nature and needs (Kasser and Ryan, 1996).

Goals are highly relevant to the migration process. Indeed, migration is often set in motion when people attempt to maximize their goal potentials. The migration process has a substantial impact on the individual’s demands, challenges, opportunities, and resources; it necessitates substantial goal adjustment and the reformulation of aspirations. Whereas certain goals may need to be put on hold, other goals – goals that may not have been important in the home country – may become more urgent (Kruglanski et al., 2002) upon migration. Chirkov et al. (2007) postulated that research on the goals of migrants is not yet well developed and that contemporary motivation theories have yet to be applied to migration research. In a similar vein, we argue that applying a goal pursuit perspective to migration will help us to better understand migrants’ acculturation and subsequent well-being. Such research is scarce and in the last decade there have been only a handful studies that emphasized migrant goal pursuit (see Tóth-Bos et al., 2019).

First, we argue that the attainment of intrinsic goals may help migrants to feel adjusted to their new cultural context. Indeed, attaining intrinsic goals, such as having loving relationships or doing something for the sake of others helps migrants to adjust to the host country in different ways: It enhances migrants’ sense of belonging and most likely supports social identification with host-country nationals, which is crucial for acculturation (Ward et al., 2001). Through building a social circle, participation in a supportive community and increasing opportunities for personal growth, migrants may feel valued by the host society, and less likely continue longing for friends and practices of the home country. Successful goal attainment might even serve as a validation of the (often difficult) choice to migrate. Similarly, attaining health goals allows migrants to participate in the social context, establishing and maintaining important connections through friendships, work, and leisure activities. Pursuing and attaining personal growth or self-development goals indicates that the migrant is positively responding to personal identity challenges and is willing to evaluate self-relevant information and change accordingly (Ozer, 2017), which in turn enhances his or her functioning in the new cultural context. A couple of previous studies investigated the intrinsic goal attainment-acculturation link and these are generally in line with the propositions of self-determination theory (Deci and Ryan, 2000) and in support of our expectations (see Tartakovsky and Schwartz, 2001; Gong, 2003; Gong and Fan, 2006; Chirkov et al., 2008; Pinto et al., 2012; Zhou, 2014; Zimmermann et al., 2017; Yang et al., 2018). For instance, Yang et al. (2018) studied international students and found that when the motivation to study abroad was self-determined, students experienced less culture shock. Yehuda-Sternfeld and Mirsky (2014) showed that the lack of attainment of social belongingness goals of those Jewish individuals from the US who resided to Israel, made them feel disconnected from the host society which catalyzed their return intentions, and their actual repatriation to the US.

Second, we argue that acculturation will serve as a mediator in the relationship between intrinsic goal attainment and well-being. As we already explained in the previous, there is ample evidence showing that acculturation and well-being are positively related to each other (see Yoon et al., 2013). In addition, there is also evidence linking the attainment of intrinsic goals to well-being (Emmons, 1986; Carver and Scheier, 1990; Brunstein, 1993; Wiese and Freund, 2005; Niemiec et al., 2009). Achieving goals provides feedback to people that they are able to overcome obstacles through effort, which in turn enhances their happiness (Emmons, 1986; Niemiec et al., 2009). Only a couple of studies investigated the attainment of intrinsic goals in relation to well-being in the migrant context. For instance, it has been found that those students who where intrinsically motivated to move abroad and indeed migrated experienced greater subjective well-being (Yang et al., 2018) and were less likely to engage in self-destructive behavior (Zhang and Zhang, 2017). As such, previous research seems to support the notion that attainment of intrinsic goals may increase migrant well-being.

Notably, previous research on extrinsic goals in the migration context found them to be detrimental to various indicators of cross-cultural adjustment (see Chirkov et al., 2007, 2008; Pinto et al., 2012). While extrinsic goals (such as higher paycheck in the host-country) might be appealing for migrants, they might not be predictive of successful adjustment in a foreign culture in the long run (see Hendriks et al., 2018). In addition, extrinsic goals are generally thought to be detrimental to well-being (Kasser and Ryan, 1996) and the potential beneficial effects of extrinsic goals may be short lived and temporary (Vansteenkiste et al., 2007). For this reason, in present paper we focus solely on intrinsic goal attainment and goal importance and their benefit on acculturation.

Beneficial effects of attaining goals may be stronger for goals that are deemed important. Research on the joint effect of intrinsic goal importance and goal attainment showed that the more intrinsic (but not extrinsic) goal importance and goal attainment were in line, the more satisfied people were with their lives (Tóth et al., 2018; also see Niemiec et al., 2009). In contrast, having important goals without being able to attain them is often accompanied by a sense of longing and the feeling that needs are not fully satisfied (Mayser et al., 2008). The interplay between goal attainment and goal importance, however, has rarely been investigated in relation to acculturation. To our knowledge, only one study from Zimmermann et al. (2017) investigated whether the congruence between sojourners’ pre-departure goals (e.g., personal development) and the actual attainment of these goals affected sociocultural and psychological adjustment. They found that when the goal importance and goal attainment were in congruence, sojourners’ adjustment (and satisfaction) increased linearly. As such, we posit that the beneficial effect of intrinsic goal attainment on acculturation is stronger when these goals are also deemed personally important.

In sum, we aim to (1) test the role of acculturation in the relationship between intrinsic goal attainment and migrant well-being and (2) investigate the interplay of intrinsic goal importance and goal attainment on acculturation. Based on the above, our hypotheses are as follows (see Figure 1):

Hypothesis 1*a*: The positive relationship between migrant intrinsic goal attainment and migrant satisfaction with life is mediated by migrant acculturation level.

*Hypothesis 1b*: The negative relationship between migrant intrinsic goal attainment and migrant anxiety and depression is mediated by migrant acculturation level.

*Hypothesis 2*: The relationship between intrinsic goal attainment and acculturation is moderated by intrinsic goal importance, such that this relationship is stronger to the extent that goals are perceived to be important.

## Overview of the Studies

In two experimental studies and two online surveys we tested the proposed relationships between goal attainment, goal importance, acculturation, and well-being. Study 1 was a scenario study describing the life of a migrant (called Mia). We manipulated both the level of intrinsic goal attainment (high vs. low) and the level of intrinsic goal importance (high vs. low). After reading the scenario text, participants filled in a questionnaire assessing their perceptions of Mia’s level of acculturation, anxiety and life-satisfaction. In Study 2a we aimed to replicate Study 1, but this time we only manipulated the level of goal attainment (high vs. low) and we measured perceived goal importance. In Study 2b and Study 3 we used self-report questionnaires to test the proposed relationships. In addition, whereas Study 1 relied on a US non-migrant sample (assessing perceptions of migrants by non-migrants), Study 2a, Study 2b, and Study 3 relied on a sample of first-generation self-initiated migrants^1^. All study procedures have been approved by the ethics committee of the university, and we obtained informed consent from all participants.

## Method Study 1

### Participants and Design

A total of 423 U.S. citizens with a predominantly non-migrant background (only 3% of them were born outside of the United States) participated in an online scenario experiment. After screening out respondents who failed any of the three attention checks, 395 people remained in the final analysis (239 male, *M*_age_ = 35.5, *SD* = 10.47). The respondents were randomly assigned to a 2 (intrinsic goal attainment: high vs. low) × 2 (intrinsic goal importance: high vs. low) between-subjects design, forming four conditions: low intrinsic goal importance – low intrinsic goal attainment (*n* = 104); low importance – high attainment (*n* = 102); high importance – low attainment (*n* = 102); and high importance – high attainment (*n* = 87). We recruited respondents on Amazon’s Mechanical Turk (MTurk) platform and paid them 1 USD for their participation. Note that previous research has shown that data obtained via MTurk are as reliable as those obtained via traditional methods (Paolacci et al., 2010; Cheung et al., 2017; Buhrmester et al., 2018).

### Procedure and Manipulation

After answering some questions pertaining to demographic details, participants were informed that they would read a description of the life of a migrant called Mia. In the introduction to the scenario we made clear that Mia was not born in the United States, but we did not specify the length of her stay in the country. We highlighted that she moved to the United States voluntarily (i.e., “She decided to move to the US a while ago…”) and that she can return to the country of origin whenever she wants (i.e., she is not a refugee). To manipulate intrinsic goal importance and attainment we relied on the items of the Aspiration Index questionnaire (Kasser and Ryan, 1996) that focuses on four intrinsic goals, namely relationships, growth, community, and health.

In the first part of the text, we manipulated the level of the importance of these goals. In the *high importance* condition, the goals were presented as being relevant and motivating to Mia. For example, “Mia has a few main goals in life that she also kept pursuing after her arrival in the US. She always wanted to have deep enduring relationships. She feels that it is very important to develop and learn new things. She wants to make the world a better place. She wants to stay healthy…” In the *low importance* condition, the goals were described as more trivial and insignificant to Mia. For example, “Mia never had particular main goals in life, also not after her arrival to the US. She has never been too interested in having deep, enduring relationships. She also does not seem to care much about personal development and learning new things. It is not so important to her that she wants to have a particularly healthy diet or plan regular exercise.”

In the second part of the text, participants read about the extent to which Mia attained these goals. In the *high attainment* condition the participants read, for instance, “She has loving relationships. She has developed a fair amount of insight into who she is as a person. She is involved in community work. She has a healthy lifestyle.” In the *low attainment* condition, participants read, for instance, “She doesn’t have many loving relationships, or friends she can count on. She is lacking insight into who she is as a person. She does not have a healthy lifestyle…”

At the end of the text, participants were asked to respond to items comprising our manipulation checks and main dependent variables after which they were thanked for their participation.

### Dependent Measures

#### Manipulation Checks

To assess the success of the manipulation of intrinsic goal importance, participants were presented with the 4 intrinsic goals (e.g., having loving relationships, living a healthy lifestyle) and asked whether these goals were important to Mia (*yes* or *no*). To assess the manipulation of attainment we presented the same set of goals to the participants and asked whether Mia attained those goals (*yes* or *no*).

#### Acculturation

To measure the perceived degree of Mia’s acculturation we used a composite 17-item scale (α = 0.92) measuring psychological as well as sociocultural adaptation. We used items of the Psychological Adaptation Scale (BPAS; Demes and Geeraert, 2014) and added 7 items covering various aspects of sociocultural adaptation, such as social skills, culture learning, and behavioral competence (as indicated in Searle and Ward, 1990; Ward and Kennedy, 1999; Demes and Geeraert, 2014). The items were stated in the third-person perspective to reflect the participants’ perspective on Mia’s acculturation. For example, “Mia felt… excited about being in the US”; “Mia felt… sad to be away from home country”; “I think Mia fits in the US culture”; and “I think Mia understands how things are done in the US.” Respondents rated how strongly they agreed with each statement on a scale of 1 (*strongly disagree*) to 7 (*strongly agree*).

#### Life Satisfaction

We used the Satisfaction with Life Scale (SWLS; Diener et al., 1985) to measure participants’ perception of Mia’s life satisfaction. The five items of the scale (α = 0.96), were adapted to capture the participants’ third-person perspective (e.g., “In most ways her life is close to ideal”). Participants rated how strongly they agreed with each statement on a scale of 1 (*strongly disagree*) to 7 (*strongly agree*).

#### Anxiety

We used the GAD-7 scale (Spitzer et al., 2006), a seven-item assessment for generalized anxiety disorder (α = 0.95), to measure the degree of Mia’s perceived level of anxiety. We modified the scale items to reflect third-person perspective, asking participants to rate how often they thought Mia had experienced certain problems in the last 2 weeks (e.g., “feeling nervous, anxious, or on edge” or “worrying too much about different things*”*) on a scale from 1 (*never*) to 4 (*nearly every time*).

## Results Study 1

### Manipulation Checks

To assess whether our manipulations were successful, we first conducted a χ^2^-test on participants’ answers to the goal importance questions. A total of 72% of participants answered three out of four manipulation check questions correctly in the low importance condition, and 90% did so in the high importance condition, χ^2^(4) = 218.52, *p* < 0.01. On the manipulation check questions of goal attainment, 87% of participants answered at least three out of four questions correctly in the low goal attainment condition, and 88% in the high goal attainment condition, χ^2^(4) = 269.73, *p* < 0.01. We conclude that the manipulation was sufficiently successful.

### Hypothesis Testing

To test our models and hypotheses we conducted regression analyses using the Hayes’ (2018) Process macro in SPSS (model 7). We used intrinsic goal attainment as the predictor variable, life satisfaction and anxiety as the dependent variables, and perceived acculturation as the mediator. Intrinsic goal importance was added as moderator of the intrinsic goal attainment – acculturation relationship^2^.

**FIGURE 2:**
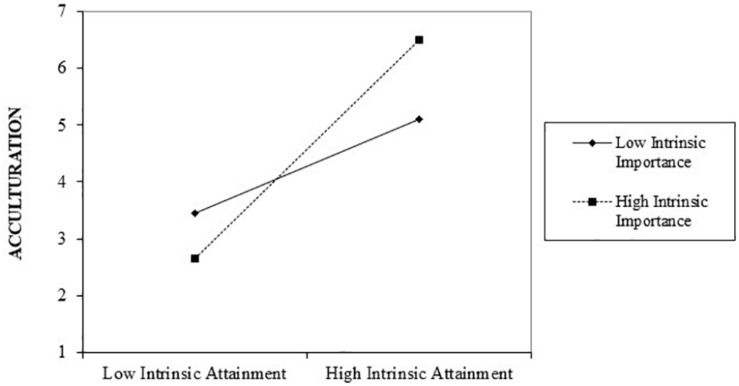
Acculturation as a function of intrinsic goal attainment and goal importance (±1 SD).

The moderated mediation analysis (see Table 1) revealed a significant positive main effect of goal attainment on Mia’s perceived acculturation (*b* = 1.38, *p* < 0.01) and on life satisfaction (*b* = 1.67, *p* < 0.01) and a significant negative relationship between goal attainment and perceived anxiety (*b* = −0.28, *p* < 0.01). We also found a significant positive relationship between Mia’s perceived acculturation and life satisfaction (*b* = 0.78, *p* < 0.01) and a significant negative relationship between Mia’s perceived acculturation and anxiety (*b* = −0.38, *p* < 0.01). Moreover, we tested the indirect effect of intrinsic goal attainment on life satisfaction and anxiety via acculturation. Consistent with Hypothesis 1a, acculturation emerged as a significant mediator for the effect of intrinsic goal attainment on life satisfaction when importance was high (*index* = 1.30, 95% *CI* [1.03, 1.59]) and low (*index* = 0.87, 95% *CI* [0.66, 1.09]). Consistent with Hypothesis 1b, acculturation mediated the relationship between intrinsic goal attainment and anxiety again both when importance was high (*index* = 0.64, *CI* [−0.80, −0.50]) and low (*index* = −0.43, 95% *CI* [−0.57, −0.30]). We also found a significant interaction effect between goal attainment and goal importance on perceived acculturation (*b* = 0.55, *p* < 0.01), supporting Hypothesis 2. The positive relationship between intrinsic goal attainment and acculturation appeared to be stronger when those goals were believed to be more important (see Table 1 and Figure 2) rather than less important.

**TABLE 1:** Model estimation results in Study 1 for assessing moderated mediation wherein intrinsic goal attainment and intrinsic goal importance interact to influence life satisfaction (SWLS) and anxiety (GAD-7) through acculturation.

	**Mediator variable model (DV = Acculturation)**				
**Predictor**	***B***	***SE***	***t*(378)**	***LLCI***	***ULCI***
Constant	4.42	0.04	96.43**	4.33	4.51
Intrinsic goal attainment	1.38	0.09	14.99**	1.19	1.56
Intrinsic goal importance	0.16	0.09	1.73	–0.02	0.34
Int.Goal Att. × Int.Goal Imp.	0.55	0.18	3.01**	0.19	0.91

		**Conditional effect of the predictor** **at values of the moderator**			
	**Index**		**SE**	**LLCI**	**ULCI**

Acculturation if importance low	1.11		0.12	0.86	1.36
Acculturation if importance high	1.67		0.13	1.40	1.93

	**Dependent variable model (DV = SWLS)**				
**Predictor**	***B***	***SE***	***t*(378)**	***LLCI***	***ULCI***
Constant	0.61	0.25	2.35*	0.10	0.12
Acculturation	0.78	0.06	13.56**	1.40	1.91
Intrinsic goal attainment	1.66	0.13	12.72**	0.66	0.89

	**Conditional indirect effects of the**				
	**predictor at values of the moderator**				
	***Index***		***Boot SE***	***LLCI***	***ULCI***

SWLS if goal importance low	0.87		0.11	0.66	1.09
SWLS if goal importance high	1.30		0.14	1.03	1.59

	**Dependent variable model (DV = GAD-7)**				
**Predictor**	***B***	***SE***	***t*(378)**	***LLCI***	***ULCI***
Constant	3.50	0.16	22.04**	3.19	3.82
Acculturation	–0.38	0.03	−3.61**	–0.45	–0.31
Intrinsic goal attainment	–0.28	0.08	−10.91**	–0.44	–0.13

	**Conditional indirect effects of the**				
	**predictor at values of the moderator**				
	***Index***		***Boot SE***	***LLCI***	***ULCI***

GAD-7 if goal importance low	–0.43		0.06	–0.57	–0.30
GAD-7 if goal importance high	–0.64		0.07	–0.80	–0.50

*p < 0.05; **p < 0.01 (two-tailed significance).

## Method Study 2 (2A and 2B)

### Participants and Design

For Study 2 we asked first generation migrants living in the United Kingdom to participate. The respondents participated in two different studies: A scenario study assessing perceptions of a described migrant (Study 2a) and a survey assessing respondents own experiences (Study 2b). Given that the entire questionnaire was in English, which is not the mother tongue of the participants, the attention check questions also served as language check.

A total of 334 first-generation migrants participated in Study 2a. Again, people who failed any of the three attention checks were screened out, leaving a total sample of 311 participants (219 female, *M*_age_ = 34.4, *SD* = 10.36). The participants were randomly assigned to the high (*n* = 152) or low (*n* = 159) intrinsic goal attainment condition, and a perceived intrinsic goal importance score was added to the design as a continuous variable. Participants were of Central and Eastern European origin (e.g., 58% from Poland, 11% from Hungary, 6% from Czech Republic). On average, participants had been living in the United Kingdom for nearly 10 years (*M* = 9.43, *SD* = 6.30). Half of the participants had lived in a foreign country outside of their home country before moving to the United Kingdom. Fifty-seven percent of the participants had obtained a college degree or higher, and 84% had a paid job at the time.

Study 2b consisted of 290 people. After screening out two extreme outliers the final sample was *N* = 288 (71% female, *M*_age_ = 34.52 *SD* = 10.32). Note that the sample size for Study 2b is somewhat lower than the sample size for Study 2a because we could only include those respondents who answered the questions pertaining to the predictor and dependent variables for this study.

### Procedures

We recruited respondents on the Qualtrics Panel platform and paid them 12 USD for their participation. Qualtrics Panel rigorously monitors data quality (see Qualtrics, 2018) and is considered a highly reliable online sampling source (Roulin, 2015).

Participating migrants first filled out some demographic questions and questions relating to their own well-being and acculturation. They were then introduced to the scenario experiment (Study 2a). Prior to reading the scenario we asked the respondents’ opinion about how important they thought the four intrinsic goals are to Mia. Thereafter, participants read a text explaining the extent to which Mia had been able to reach those intrinsic goals, again using the items of the Aspiration Index to manipulate goal attainment (high vs. low) similar to what we did in Study 1. Participants then answered questions pertaining to Mia’s perceived acculturation and well-being (life satisfaction and anxiety), as well as some questions that served as manipulation checks.

After completing the scenario study, we asked respondents to answer some questions about their personal goal pursuit, first answering a question about which goals are important to them, then about to what extent they have attained these goals (Study 2b). We placed questions regarding their own well-being and acculturation deliberately before the scenario experiment to prevent the emergence of biased answer patterns.

### Measures Study 2a

#### Intrinsic Goal Importance

Respondents were asked to rate how important they thought certain goals might be for Mia. We presented a total of eight intrinsic goals, two for each intrinsic goal dimensions (e.g., “to grow and learn new things,” “to feel that there are people who really love her and whom she loves,” “to be free from sickness”), and asked participants to indicate their opinion on how important these goals were to Mia (α = 0.87) on a scale from 1 (*not at all important*) to 7 (*very important*).

#### Manipulation Check

To assess the success of the intrinsic goal attainment manipulation, we used the same four statements as in Study 1, but this time we asked participants to indicate to what extent they thought Mia had attained each goal on a scale from 1 (*not at all*) to 7 (*completely*).

#### Acculturation

We used the same composite scale of psychological- (BPAS), and sociocultural adaptation (BSAS) as in Study 1 (α = 0.94).

#### Life Satisfaction

As in Study 1, we administered the SWLS (α = 0.97).

#### Anxiety

As in Study 1, we used the GAD-7 to measure respondents’ perception of Mia’s anxiety level (α = 0.96).

## Results Study 2A

### Manipulation Check

Testifying to the successfulness of our manipulation, an independent sample *t*-test showed that respondents in the low attainment condition rated Mia’s goal attainment significantly lower (*M* = 2.45, *SD* = 1.49) than did respondents in the high attainment condition (*M* = 6.23, *SD* = 0.75), *t*(236) = −28.327, *p* < 0.01.

### Hypothesis Testing

To test our hypotheses, we again relied on Hayes’ (2018) Process macro in SPSS (model 7). We used the same predictor-, dependent-, and moderator variables as in Study 1^2^.

The analysis (see Table 2) revealed significant positive relationships between goal attainment and Mia’s perceived acculturation and (*b* = 2.23, *p* < 0.01) life satisfaction (*b* = 1.60, *p* < 0.01) and a significant negative relationship between goal attainment and perceived anxiety (*b* = −0.32, *p* < 0.01). We also found that Mia’s perceived acculturation positively predicted perceived life satisfaction (*b* = 0.79, *p* < 0.01) and negatively predicted perceived anxiety (*b* = −0.41, *p* < 0.01). In line with Hypothesis 1a, acculturation mediated the effect of intrinsic goal attainment on life satisfaction at high (*index* = 1.78, 95% *CI* [1.41, 2.15]) and low (*index* = 1.77, 95% *CI* [1.42, 2.13]) values of goal importance. Similarly, acculturation mediated the relationship between intrinsic goal attainment and anxiety when importance was high (*index* = −0.92, 95% *CI* [−1.14, −0.71]) and low (*index* = −0.91, 95% *CI* [−1.08, −0.74]), giving support to Hypothesis 1b. In contrast to Hypothesis 2, we did not find a significant interaction effect between goal attainment and goal importance on perceived acculturation (see Table 2).

**TABLE 2:** Model estimation results in Study 2a for assessing moderated mediation wherein intrinsic goal attainment and intrinsic goal importance interact to influence life satisfaction (SWLS) and anxiety (GAD-7) through acculturation.

	**Mediator variable model (DV = Acculturation)**				
**Predictor**	***B***	***SE***	***t*(306)**	***LLCI***	***ULCI***
Constant	4.29	0.05	83.16**	4.19	4.39
Intrinsic goal attainment	2.23	0.10	21.58**	2.02	2.43
Intrinsic goal importance	0.07	0.05	1.28	–0.37	0.18
Int.Goal Att. × Int.Goal Imp.	0.00	0.11	0.05	–0.21	0.22

	**Dependent variable model (DV = SWLS)**				
**Predictor**	***B***	***SE***	***t*(306)**	***LLCI***	***ULCI***

Constant	0.56	0.23	2.36*	0.09	1.03
Acculturation	0.79	0.05	14.60**	0.68	0.90
Intrinsic goal attainment	1.60	0.15	10.26**	1.29	1.91

	**Conditional indirect effects of the**				
	**predictor at values of the moderator**				
	***Index***		***Boot SE***	***LLCI***	***ULCI***

SWLS if goal importance low	1.77		0.18	1.42	2.13
SWLS if goal importance high	1.78		0.19	1.41	2.15

	**Dependent variable model (DV = GAD-7)**				
**Predictor**	***B***	***SE***	***t*(306)**	***LLCI***	***ULCI***

Constant	3.88	0.15	24.66**	3.57	4.19
Acculturation	–0.41	0.03	−11.41**	–0.48	–0.33
Intrinsic goal attainment	–0.32	0.10	−3.17**	–0.52	–0.12

	**Conditional indirect effects of the**				
	**predictor at values of the moderator**				
	***Index***		***Boot SE***	***LLCI***	***ULCI***

GAD-7 if goal importance low	–0.91		0.08	–1.08	–0.74
GAD-7 if goal importance high	–0.92		0.10	–1.14	–0.71

*p < 0.05; **p < 0.01 (two-tailed significance).

### Measures Study 2b

#### The Importance of Self-Set Goals

We asked all respondents to list three of their current goals in life and to rate their importance on a scale from 1 (*not at all important*) to 7 (*very important*). We then computed the average self-set goal importance score. The Cronbach α for the self-set goal importance scale is relatively low (α = 0.60), perhaps due to our method for assessing self-set goal importance. Respondents were asked to list their main life goals using full sentences and then to rate each goal’s importance. Each sentence had to respectively start with “I want to…”; “My goal is to.”; and “I aspire to….” Cronbach α may be suppressed due to the qualitative nature of the question (Shenton, 2004) as well as due to the three different beginnings of the sentence. The Cronbach α of short questionnaires is often lower than the 0.7 cutoff point (Wanous et al., 1997; Wanous and Hudy, 2001; Tavakol and Dennick, 2011).

#### The Attainment of Self-Set Goals

After indicating the importance of their three goals, respondents rated the extent to which they had attained each goal on a scale from 1 (*not at all*) to 7 (v*ery much*). We then computed the average self-set goal attainment score (α = 0.70).

#### Acculturation

We used a composite scale of the psychological (BPAS) and sociocultural (BSAS) adjustment scales by Demes and Geeraert (2014). Respondents rated the extent to which they agreed with each statement (e.g., feeling “… excited about being in the United Kingdom” or “…sad to be away from home country”) on a scale from 1 (*strongly disagree*) to 7 (*strongly agree*). Respondents also indicated how difficult they found it to adapt to certain situations in the United Kingdom (e.g., climate, food and eating, social environment) on a scale from 1 (*very difficult*) to 7 (*very easy*). The average score of our 22 acculturation items (α = 0.86) was calculated and served as our acculturation measure.

#### Life Satisfaction

As in the previous studies we used the SWLS (Diener et al., 1985), this time as a self-report measure (α = 0.90): Respondents indicated the extent to which they agreed with each statement on a scale from 1 (*not at all) to* 7 (*completely*).

#### Anxiety

As in the previous studies, we used the GAD-7 (Spitzer et al., 2006), this time as a self-report measure (α = 0.91): We asked respondents to indicate how often they felt a certain way in the last 2 weeks on a scale from 1 (*never*) to 4 (*nearly every time*).

## Results Study 2B

### Preliminary Analyses

Descriptives and intercorrelations of the study variables are provided in Table 3.

**TABLE 3:** Descriptive statistics and intercorrelations of the study variables in Study 2b.

**Variables**	**Mean**	**SD**	**1.**	**2.**	**3.**	**4.**
1. SWLS	4.19	1.36				
2. GAD-7	1.98	0.72	−0.40**			
3. Acculturation	5.00	0.86	0.29**	−0.31**		
4. Self-set goal importance	6.47	0.67	−0.02	−0.06	−0.02	
5. Self-set goal attainment	3.79	1.37	0.46**	−0.33**	0.23**	0.14*

*p < 0.05; **p < 0.01 (two-tailed significance).

Self-set goal attainment was related positively to acculturation (*r* = 0.23, *p* < 0.01) and life satisfaction (*r* = 0.46, *p* < 0.01), and negatively to anxiety (*r* = −33, *p* = 0.02). The respondents’ own acculturation showed significant associations with life satisfaction (*r* = 0.29, *p* < 0.01) and anxiety (*r* = −0.31, *p* < 0.01) in the expected directions.

### Hypothesis Testing

**FIGURE 3:**
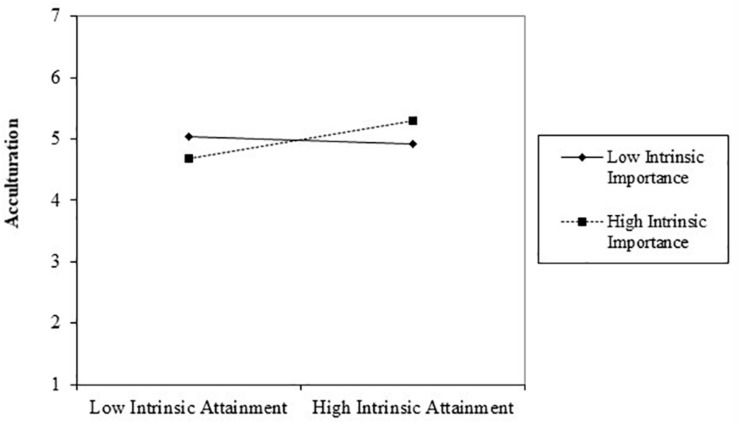
Acculturation as a function of intrinsic goal attainment in Study 2b.

Once again, we tested our hypotheses by using Hayes’ Process macro (Process Model 7) to conduct moderated mediation analysis. We used the same predictor-, dependent-, and moderator variables as in Study 1^2^. The results (see Table 4) revealed a significant positive main effect of self-set goal attainment on acculturation (*b* = 0.13, *p* < 0.01) and life satisfaction (*b* = 0.41, *p* < 0.01) and a significant negative main effect of goal attainment on perceived anxiety (*b* = −0.15, *p* < 0.01). We furthermore found a significant positive relationship between perceived acculturation and life satisfaction (*b* = 0.32, *p* < 0.01) and a significant negative relationship between perceived acculturation and anxiety (*b* = −0.21, *p* < 0.01). We tested the indirect effect of intrinsic goal attainment on life satisfaction and on anxiety via acculturation. Consistent with Hypothesis 1a, acculturation emerged as significant mediator for the effect of intrinsic goal attainment on life satisfaction when goal importance was high (*index* = 0.07, 95% *CI* [0.02, 0.11]) but not when importance was low. Acculturation also mediated the relationship between intrinsic goal attainment and anxiety but only if the goal was considered to be highly important (*index* = −0.04, 95% *CI* [0.07, −0.02]), and not when goal importance was low, giving support to Hypothesis 1b. Hypothesis 2 was confirmed, as we found a significant interaction between self-set goal attainment and importance on acculturation (*b* = 0.17, *p* = 0.02). The results indicate that goal attainment positively predicts acculturation to the extent that the goals are deemed important (see Figure 3).

**TABLE 4:** Model estimation results in Study 2b for assessing moderated mediation wherein self-set goal attainment and self-set goal importance interact to influence life satisfaction (SWLS) and anxiety (GAD-7) through acculturation.

	**Mediator variable model (DV = Acculturation)**				
**Predictor**	***B***	***SE***	***t*(288)**	***LLCI***	***ULCI***
Constant	4.99	0.05	99.58**	4.90	5.09
Intrinsic goal attainment	0.13	0.03	3.42**	0.05	0.20
Intrinsic Goal Importance	–0.03	0.08	–0.41	–0.20	0.13
Int.GoalAtt. × Int.GoalImp.	0.17	0.07	2.36*	0.02	0.31

		**Conditional effect of the predictor**				
	**at values of the moderator**				
	***Effect***		***Boot SE***	***LLCI***	***ULCI***

Acculturation if importance low	0.04		0.05	−0.07	0.16
Acculturation if importance high	0.21		0.04	0.12	0.30

	**Dependent variable model (DV = SWLS)**				
**Predictor**	***B***	***SE***	***t*(288)**	***LLCI***	***ULCI***

Constant	2.56	0.42	5.98**	1.71	3.40
Intrinsic goal attainment	0.42	0.05	7.91**	0.31	0.52
Acculturation	0.32	0.08	3.84**	0.15	0.48

	**Conditional indirect effects at**				
	**values of the moderator**				
	***Effect***		***Boot SE***	***LLCI***	***ULCI***

SWLS if importance low	0.01		0.02	−0.02	0.05
SWLS if importance high	0.07		0.02	0.03	0.11

	**Dependent variable model (DV = GAD-7)**				
**Predictor**	***B***	***SE***	***t*(288)**	***LLCI***	***ULCI***

Constant	3.05	0.23	12.94**	2.59	3.52
Intrinsic goal attainment	–0.15	0.03	−5.27**	–0.21	–0.09
Acculturation	–0.21	0.04	−4.59**	–0.30	–0.12

	**Conditional indirect effects at**				
	**values of the moderator**				
	***Effect***		***Boot SE***	***LLCI***	***ULCI***

GAD-7 if importance low	–0.01		0.01	–0.03	0.01
GAD-7 importance high	0.04		0.01	–0.07	–0.02

*p < 0.05; **p < 0.01 (two-tailed significance).

## Method Study 3

### Participants

The sample consisted of 549 Hungarian nationals who were at least 18 years old and living in the Netherlands with no predetermined end of stay. Prior to conducting our analysis, we filtered out seven extreme outliers leaving us with a final sample of 542 respondents. Of the respondents 67% were women and the average age was 35.5 years (*SD* = 10.23), which is comparable with the median age of Hungarians registered in the Dutch national statistics (Central Bureau voor de Statistiek [CBS], 2016). Forty percent of the respondents had been living in the country for 5 years or more and 28% for less than 2 years. The respondents were relatively highly educated with 67% having a college or bachelor’s degree or higher. More than 80% obtained their highest education diploma in Hungary, indicating that a large part of the migrants’ socialization happened in the country of origin. Seventy-seven percent of the respondents indicated that they had a job, of which 42% felt their job was below their qualification level. Half of the respondents indicated having full-time employment, 11% had part-time employment, and 12% were entrepreneurs or self-employed. The rest of the respondents were mainly students, women on maternity leave, or retirees. Forty-three percent of the respondents had experience living abroad before coming to the Netherlands. The majority of the respondents (65%) indicated that they would consider moving back to Hungary at some point in time, 23% planned never to go back, and 12% planned to move back in a few years.

### Procedure

We recruited the respondents using different channels, all with the help of online mediums. Several Hungarian (formal and informal) associations were asked to distribute the link to the questionnaire. Moreover, various people with widespread connections to Hungarian communities and migrant populations volunteered to help promote the questionnaire by distributing the link to the survey. As an example, during the recruitment period, a documentary (*Menjek/Maradjak* – *To leave or To Stay*) -aimed at giving an insight into the lives of Hungarian migrants in the Netherlands- was promoted in Hungary and in the Netherlands. We added the link of the survey to the promotional material of the movie premiere (e.g., via online articles, blog posts) and requested Hungarians living in the Netherlands to fill out the questionnaire.

Respondents were asked to complete an online survey designed to capture their life in the Netherlands. The entire questionnaire was in Hungarian. The first part of the survey contained detailed demographic questions regarding the respondents’ current life situation in the host country, as well as the respondents’ circumstances preceding the move from Hungary. The second part included the measurement of goal importance and goal attainment as well as measures of life satisfaction and anxiety. The third part of the questionnaire contained an evaluation of the extent of cultural adaptation, both social and psychological. Participation was voluntary and anonymous; there was no compensation for participation.

### Measures

#### Intrinsic Goal Attainment

To measure the attainment of intrinsic life goals, we used the Hungarian version of Kasser and Ryan’s (1996) Aspiration Index (Komlósi et al., 2006). Respondents were presented with a total of 20 goals (5 items per each of the 4 subscales) and asked to indicate the extent to which they had attained each goal on a scale from 1 (*not at all*) to 7 (*very much*). We calculated the intrinsic goal attainment score by averaging the score on each of the items (α = 0.90).

#### Intrinsic Goal Importance

We also used the Aspiration Index to measure intrinsic goal importance. Respondents were asked to indicate how important they considered each of the 20 goals to be on a scale from 1 (*not at all*) to 7 (*very much*). We calculated the intrinsic goal importance score by averaging the score on each of the items (α = 0.85).

#### Acculturation

We used the same composite scale of psychological- (BPAS), and sociocultural adaptation (BSAS) as in Study 2b (α = 0.89).

#### Life Satisfaction

We used the Hungarian translation (Martos et al., 2014) of the SWLS (Diener et al., 1985) to assess subjective well-being (α = 0.86). Respondents indicated their agreement with each item on a scale from 1 (*not at all*) to 7 (*completely*).

#### Depression

We used the Hungarian version (Novák et al., 2010) of the 20-item CES-D scale (Radloff, 1977). Respondents were asked to indicate how often they felt depressive symptoms (e.g., “I had crying spells,” “I could not get going,” “I was bothered by things that usually don‘t bother me”) within the past week on a scale from 0 (*rarely or none of the time*) to 3 (*most or all the time*). Notably, this scale is also considered to be well suited for testing generalized anxiety (Breslau, 1985). The reliability of the scale was α = 0.90.

## Results Study 3

### Preliminary Analyses

Descriptives and intercorrelations of the study variables are provided in Table 5. In line with our hypotheses we found a significant positive correlation between acculturation and life satisfaction (*r* = 0.44, *p* < 0.01) and a negative correlation between acculturation and anxiety (*r* = −0.54, *p* < 0.01). Furthermore, intrinsic goal attainment had a positive correlation with life satisfaction (*r* = 0.49, *p* < 0.01) and acculturation (*r* = 0.33, *p* < 0.01) and a negative correlation with anxiety (*r* = −0.44, *p* < 0.01).

**TABLE 5:** Descriptive statistics and intercorrelations of the study variables in Study 3.

**Variables**	**Mean**	**SD**	**1.**	**2.**	**3.**	**4.**
1. SWLS	5.19	1.16				
2. CES-D	1.58	0.45	−0.59**			
3. Acculturation	4.92	0.86	0.44**	−0.54**		
4. Intrinsic goal importance	6.12	0.60	0.07	–0.02	–0.06	
5. Intrinsic goal attainment	5.05	0.91	0.49**	−0.44**	0.33**	0.39**

*p < 0.05; **p < 0.01 (two-tailed significance).

### Hypothesis Testing

To test our model (see Figure 1) and hypotheses we conducted regression analysis for which we relied on the Hayes’ Process macro in SPSS (Process model 7)^2^. We used the same predictor-, independent-, and moderator variables as in Study 1.

The results revealed (see Table 6) a significant main effect of intrinsic goal attainment on acculturation (*b* = 0.37, *p* < 0.01) and on life satisfaction (*b* = 0.50, *p* < 0.01), and a negative main effect on anxiety (*b* = −0.14, *p* < 0.01). Interestingly, a significant negative main effect of goal importance on acculturation (*b* = −0.25, *p* < 0.01) was unveiled. We found a significant positive relationship between acculturation and life satisfaction (*b* = 0.42, *p* < 0.01) and a significant negative relationship between acculturation and anxiety (*b* = −0.23, *p* < 0.01). Supportive of Hypothesis 1a, acculturation emerged as significant mediator for the effect of intrinsic goal attainment on life satisfaction when importance was high (*index* = 0.13, 95% *CI* [0.07, 0.20]) and low (*index* = 0.18, 95% *CI* [0.11, 0.25]). Similarly, acculturation mediated the effects of the predictors on depression at high (*index* = −0.07, 95% *CI* [−0.10, −0.04]) and low values of the moderator (*index* = −0.09, 95% *CI* [−0.13, −0.07]). We found no interaction effect between goal attainment and goal importance on acculturation, disconfirming Hypothesis 2.

**TABLE 6:** Model estimation results in Study 3 for assessing moderated mediation wherein intrinsic goal attainment and intrinsic goal importance interact to influence life satisfaction (SWLS) and depression (CES-D) through acculturation.

	**Mediator variable model (DV = Acculturation)**				
**Predictor**	***B***	***SE***	***t*(542)**	***LLCI***	***ULCI***
Constant	4.94	0.03	133.57**	4.87	5.02
Intrinsic goal attainment	0.37	0.04	9.03**	0.29	0.45
Intrinsic goal importance	–0.25	0.06	−3.87**	–0.37	–0.12
Int.GoalAtt. × Int.GoalImp.	–0.09	0.06	−1.53**	–0.21	–0.02

	**Dependent variable model (DV = SWLS)**				
**Predictor**	***B***	***SE***	***t*(542)**	***LLCI***	***ULCI***

Constant	3.12	0.25	12.39**	2.63	3.62
Intrinsic goal attainment	0.50	0.05	10.46**	0.41	0.59
Acculturation	0.42	0.05	8.31**	0.32	0.51

	**Conditional indirect effects of the**				
	**predictor at values of the moderator**				
	***Effect***		***Boot SE***	***LLCI***	***ULCI***

SWLS if importance low	0.18		0.03	0.11	0.25
SWLS if importance high	0.13		0.03	0.07	0.20

	**Dependent variable model (DV = CESD-D)**				
**Predictor**	***B***	***SE***	***t*(542)**	***LLCI***	***ULCI***

Constant	2.71	0.09	28.81**	2.53	2.9
Intrinsic goal attainment	–0.14	0.02	−8.17**	–0.18	–0.11
Acculturation	–0.23	0.02	−12.14**	–0.26	–0.19

	**Conditional indirect effects of the**				
	**predictor at values of the moderator**				
	***Effect***		***Boot SE***	***LLCI***	***ULCI***

CES-D if importance low	–0.09		0.01	–0.13	–0.07
CES-D if importance high	–0.07		0.01	–0.10	–0.04

**p < 0.01 (two-tailed significance).

## General Discussion

The beneficial effects of intrinsic goal pursuit on well-being are well established (Diener, 1984; Freund and Baltes, 2002; Wiese and Freund, 2005; Niemiec et al., 2009), but little is known about whether the attainment of important goals contributes to migrants’ acculturation. In our paper we set out to investigate whether goal attainment predicts well-being via acculturation. Furthermore, we aimed to test whether acculturation is the function of the interaction between goal attainment and goal importance. We proposed that by feeling acculturated upon realizing important goals, migrants will feel more satisfied with their lives and less anxious and depressed. Through two scenario experiments (Study 1 and Study 2a) and two online surveys (Study 2b and Study 3), we found that the attainment of intrinsic goals is positively related to (perceived) acculturation and life satisfaction and negatively to (perceived) anxiety (Study 1, 2a and 2b) and depression (Study 3). In all four studies the mediating role of acculturation in the goal attainment – well-being relationship was confirmed, supporting our first hypothesis (Hypotheses 1a and 1b).

Apparently attaining goals that support innate needs such as autonomy, competence, and connectedness (Deci and Ryan, 2000; Sheldon et al., 2004) helps migrants to fit into a new culture and to deal with the challenges of a culture change. Specifically, pursuing and attaining intrinsic goals seems to serve migration success by increasing migrants’ satisfaction with life and decreasing feelings of ill-being because attaining intrinsic goals fosters acculturation. However, in only two of the studies (Study 1 and Study 2b) we found support for the moderating role of goal importance in the relationship between goal attainment and acculturation (see Hypothesis 2). As such, we found no persistent evidence for the idea that particularly reaching important goals fosters acculturation. Indeed, it seems that sometimes the mere attainment of goals, even less important ones, could benefit migration success. Perhaps the importance of goals is only relevant in the goal attainment-well-being relationship under certain conditions. Future research may consider investigating when goal importance does or does not strengthen the effect of goal attainment on well-being.

### Strengths, Limitations, and Future Directions

In present paper we have established the relationship between goal pursuit and migrant well-being and identified acculturation as a potential underlying mechanism in this relationship. We found evidence for the proposed relationship between goal attainment, acculturation and well-being in four studies and various migrant samples, which testifies the robustness of our findings. Indeed, we consider the mixed-method approach to be a strength of the present paper. Whereas experimental designs are often criticized for their limited external validity, cross-sectional surveys are often criticized for their limited internal validity (Houdek, 2017). By using both designs, we aimed to increase the overall viability of our findings. Moreover, apart from Study 1, all studies sampled working-age, self-initiated migrants. Self-initiated migrants are not easily sampled in scientific research (Dickmann and Doherty, 2008), as evidenced by their low representations in paid online sampling panels (Qualtrics, 2018). Of course, our studies also suffer from some weaknesses. For instance, in Study 1, we relied on a sample of non-migrant individuals and we asked them to reflect on how migrants may feel in a hypothetical scenario. Admittedly, these people had limited firsthand knowledge of the migrant experience. In addition, it may be that the respondents in Study 1 -all US citizens- found the described migrant more competent or similar to the self when she was able to achieve certain goals. Previous research has indicated that realizing goals is particularly important for people from more individualistic countries (see Itai, 2008). As such, Mia may have been perceived as more similar to the self when she was able to reach (important) goals. Future research may investigate if perceptions of similarity may explain some of our findings in Study 1. Another potential issue is that we worked with the same sample of migrants in Study 2a and 2b. It might be that reading about and reflecting on a hypothetical situation of another migrant had an impact on which self-set goals migrants reported later or on their own responses. Although our sampling for Study 2a and 2b may not be ideal, the questions were placed well apart from each other in order to prevent the emergence of biased answer patterns. Moreover, in all of our studies, also the experimental ones, the relationship between acculturation and well-being was correlational in nature. However, correlation is not the same as causation, and although it makes sense from a theoretical point of view that acculturation leads to well-being (instead of the other way around), this assumption needs to be further supported by clear empirical evidence. To assess a causal relationship, future studies may particularly consider employing longitudinal designs. So far, only a limited number of studies have done so (see f.i., Turjeman et al., 2008; Zeiders et al., 2016) and although these seem to support the existence of an acculturation-well-being link more support is welcome. Also note that for assessing causality in this relationship longitudinal designs seem most suitable; another option may be to design experiments but it may be difficult to manipulate acculturation in an experimental setting.

In the current research, we focused on one specific migrant group (self-initiated migrants) and investigated a limited number of home and host countries to increase the interpretability of the findings. Specifically, we included people of Central and Eastern European origin (e.g., from Hungary, Poland, Czech Republic) who moved voluntary to Western Europe (The Netherlands and the United Kingdom). Future studies, may broaden these geographic boundaries and include different types of migrants. Strictly controlled sampling of migrants (by geographic factors, original motivation, or demographic characteristics) would allow us to understand how migrant characteristics may affect goal pursuit processes. For instance, in our studies both receiving countries were developed, individualistic countries and -when specified- sending countries were so too. However, migration between two developed countries (North-North migration; International Organization of Migration [IOM], 2013) involves different adjustment challenges and experiences than do other directions of migration (e.g., SouthNorth migration or South-South migration). For instance, it may be that in less developed countries there is a stronger focus on safety goals. Moreover, differences in norms and values between sending and receiving countries may also affect the goal pursuit process. Therefore, future research should take into account constructs such as perceived cultural distance (Suanet and van de Vijver, 2009; Demes and Geeraert, 2014), value discrepancies (Lönnqvist et al., 2013; Rudnev, 2014), and independent vs. interdependent self-construal (Markus and Kitayama, 1991, 2003; Heine et al., 2001). Also, in the present study we focused on self-initiated migrants and future research may consider including refugees in order to gain insight into how the oftentimes limited goal pursuit opportunities of refugees affect their acculturation outcomes.

To measure goal attainment and goal importance, we relied primarily on the intrinsic goal dimension of the Aspiration Index questionnaire (Kasser and Ryan, 1996). According to theory, these goals are the ones worth striving for in order to feel happy (Schmuck et al., 2000; Sheldon et al., 2004). However, our use of the Aspiration Index may have led to an oversimplified picture of migrant goals. As migration often comes with existential challenges (Ward et al., 2001), there might be more external or even “lower-level goals” (i.e., physical, safety, or esteem motives) that migrants value, which just as strongly affect their actions. By asking migrants to describe their personal goals in Study 2b, we attempted to overcome this limitation. Further research, with more extensive self-set goal measures, would perhaps provide valuable information to further understand the pillars of migration success.

Well-being, is a complex construct, involving cognitive, affective and even somatic components (Diener, 1984; Veenhoven, 2000; Hendriks et al., 2018). To assess subjective well-being, previous research has focused mainly on affective experiences (e.g., joy, vitality, sadness) and cognitive evaluations (life satisfaction). This dual focus on affective experiences and cognitive evaluations makes sense, because they are separate factors and thus separate indicators of well-being (see Lucas et al., 1996; Hendriks and Bartram, 2019). In our studies, we included measures of both anxiety (and in one study a measure of depression) and satisfaction with life in order to offer a comprehensive perspective on migrant well-being. However, we did not include measures of momentary affect (emotions) and neither did we include measures of generalized positive affect (e.g., happiness). Future research should consider including these indicators of well-being as well, so that an even more complete picture of migrant well-being can be obtained.

Finally, in present paper we have not distinguished between certain stages of migration. Migration can be seen as a linear process that begins before people actually leave their home country, when they start making plans or start entertaining the idea of moving abroad (the pre-migration stage). The process continues after relocation to the host country (the during migration stage). Finally, there is a third stage that some, but not all, migrants go through in which they relocate to yet another foreign country or repatriate to their home country (the post-migration stages; see DaVanzo, 1976; Tabor and Milfont, 2011; Carling and Collins, 2018). Evidently, goals are changing in the face of the demands and challenges of the specific stages of migration. For instance, attaining specific career goals may seem particularly important before migrating, but upon arrival in the host country migrants may focus more on fulfilling lower-level motives (i.e., physical, or safety motives; Kruglanski et al., 2002). Similarly, self-fulfillment goals may be particularly important for migrants that recently settled in the host country, but over time these might level off and other goals, such as keeping and enhancing home culture traditions may become more prominent and may even catalyze repatriation. Future research might take the temporal aspect of migration into account and investigate how acculturation and well-being are affected by the goal pursuit process in different stages of migration (see Tóth-Bos et al., 2019).

## Conclusion and Practical Implications

In the present study we tested whether goal pursuit helps migrants adjust to their host country and live happy lives in the changed context. We posited that goal pursuit enhances well-being by contributing to successful acculturation. By helping to organize one’s efforts, determine one’s actions, and frame feedback on one’s progress, goals are important anchors for people. As the migration process is often set in motion to maximizing goal potentials, the effects of goal-directed behavior may be particularly evident for migrants. The realization of important goals gives a structure to the everyday life of migrants. Realizing goals may give migrants a sense of control that enables them to navigate their new context and manage the unknown and uncertain. It might even legitimize migrants’ choice to change countries and make them feel it is worthwhile to stay despite hardship. It might be fruitful for migrants and for people who are about to emigrate to set out realistic, tangible goals across various life domains to prevent vague and impracticable aspirations that leave them without any anchor for the course of their everyday lives. Realistic and tangible goals may be more attainable and as such affect acculturation and well-being of migrants. There is a higher need for psychological counseling among migrants as compared to non-migrants (Balkir Neftçi and Barnow, 2016). Clinicians may want to focus on helping migrants find motivating goals for which attainment is feasible and rewarding. Helping to establish realistic goals might foster migrants’ cultural adjustment, which in turn will shield them from negative thoughts and help them conduct a content life.

## Data Availability Statement

The raw data supporting the conclusions of this article will be made available by the authors, to any qualified researcher, without reservation.

## Author Contributions

AT-B took care of data collection and data analysis. AT-B and BW drafted the manuscript with the AT-B taking the lead. KF engaged in several rounds of feedback. The authors are co-responsible for developing the theoretical model and study designs.

## Conflict of Interest

The authors declare that the research was conducted in the absence of any commercial or financial relationships that could be construed as a potential conflict of interest.

## Footnotes

1 Power analysis for an ANOVA with 4 groups using an alpha of 0.05, a power of 0.80, and a medium effect size (*f* = 0.25) (Faul et al., 2013) shows that the desired sample size is 180, indicating that our sample sizes for Study 1 and 2a are large enough. Power analyses for the for the survey studies using a margin of error of 5% and a confidence level of 95% shows a desired sample size of 370 respondents. The sample size for Study 2b was 290 and for Study 3 it was 549. Sample size for Study 2b is on the low side as a result of practical constraints.

2 Including gender, age, and educational background as covariates does not change the conclusions of our findings.
